# Variants of the *FADS1 FADS2* Gene Cluster, Blood Levels of Polyunsaturated Fatty Acids and Eczema in Children within the First 2 Years of Life

**DOI:** 10.1371/journal.pone.0013261

**Published:** 2010-10-11

**Authors:** Peter Rzehak, Carel Thijs, Marie Standl, Monique Mommers, Claudia Glaser, Eugène Jansen, Norman Klopp, Gerard H. Koppelman, Paula Singmann, Dirkje S. Postma, Stefanie Sausenthaler, Pieter C. Dagnelie, Piet A. van den Brandt, Berthold Koletzko, Joachim Heinrich, for the KOALA and the LISA study group

**Affiliations:** 1 Helmholtz Zentrum München, German Research Center for Environmental Health, Institute of Epidemiology, Neuherberg, Germany; 2 Institute of Medical Informatics, Biometrics and Epidemiology, Chair of Epidemiology, Ludwig-Maximilians University Munich, Oberschleißheim, Germany; 3 Department of Epidemiology, CAPHRI School of Public Health and Primary Care, Maastricht University, Maastricht, The Netherlands; 4 Division of Metabolic Diseases and Nutritional Medicine, Dr von Hauner Children's Hospital, Ludwig-Maximilians University Munich, Munich, Germany; 5 Laboratory for Health Protection Research, National Institute of Public Health and the Environment, Bilthoven, The Netherlands; 6 Department of Pediatric Pulmonology and Pediatric Allergology, Beatrix Children's Hospital, University Medical Center Groningen, University of Groningen, Groningen, The Netherlands; 7 Department of Pulmonology, University Medical Center Groningen, University of Groningen, Groningen, The Netherlands; 8 Department of Epidemiology, GROW School for Oncology and Developmental Biology, Maastricht University, Maastricht, The Netherlands; Ludwig-Maximilians-Universität München, Germany

## Abstract

**Background:**

Association of genetic-variants in the *FADS1-FADS2*-gene-cluster with fatty-acid-composition in blood of adult-populations is well established. We analyze this genetic-association in two children-cohort-studies. In addition, the association between variants in the *FADS*-gene-cluster and blood-fatty-acid-composition with eczema was studied.

**Methods and Principal Findings:**

Data of two population-based-birth-cohorts in the Netherlands and Germany (KOALA, LISA) were pooled (n = 879) and analyzed by (logistic) regression regarding the mutual influence of single-nucleotide-polymorphisms (SNPs) in the *FADS*-gene-cluster (rs174545, rs174546, rs174556, rs174561, rs3834458), on polyunsaturated fatty acids (PUFA) in blood and parent-reported eczema until the age of 2 years. All SNPs were highly significantly associated with all PUFAs except for alpha-linolenic-acid and eicosapentaenoic-acid, also after correction for multiple-testing. All tested SNPs showed associations with eczema in the LISA-study, but not in the KOALA-study. None of the PUFAs was significantly associated with eczema neither in the pooled nor in the analyses stratified by study-cohort.

**Conclusions and Significance:**

PUFA-composition in young children's blood is under strong control of the *FADS*-gene-cluster. Inconsistent results were found for a link between these genetic-variants with eczema. PUFA in blood was not associated with eczema. Thus the hypothesis of an inflammatory-link between PUFA and eczema by the metabolic-pathway of LC-PUFAs as precursors for inflammatory prostaglandins and leukotrienes could not be confirmed by these data.

## Introduction

It has been well established that genetic variants in the fatty acid desaturase genes (*FADS1* and *FADS2*) associate with the fatty acid composition in adult populations [1]. Several studies have shown that the Δ-5 and the Δ-6 desaturase enzymes are involved in fatty acid metabolism in adults and that these enzymes are genetically regulated by variants of the *FADS1* and *FADS2* genes, respectively [2]–[8].

Empirical and theoretical evidence exist that fatty acid metabolism may be involved in atopic eczema [9]–[14]. Some studies found, that dietary intake of certain fatty acids can contribute to the development of allergic diseases. Kompauer et al showed a positive association between hay fever and arachidonic acid (AA) intake, and allergic sensitisation and oleic acid intake in German adults [15].

**Figure 1:**
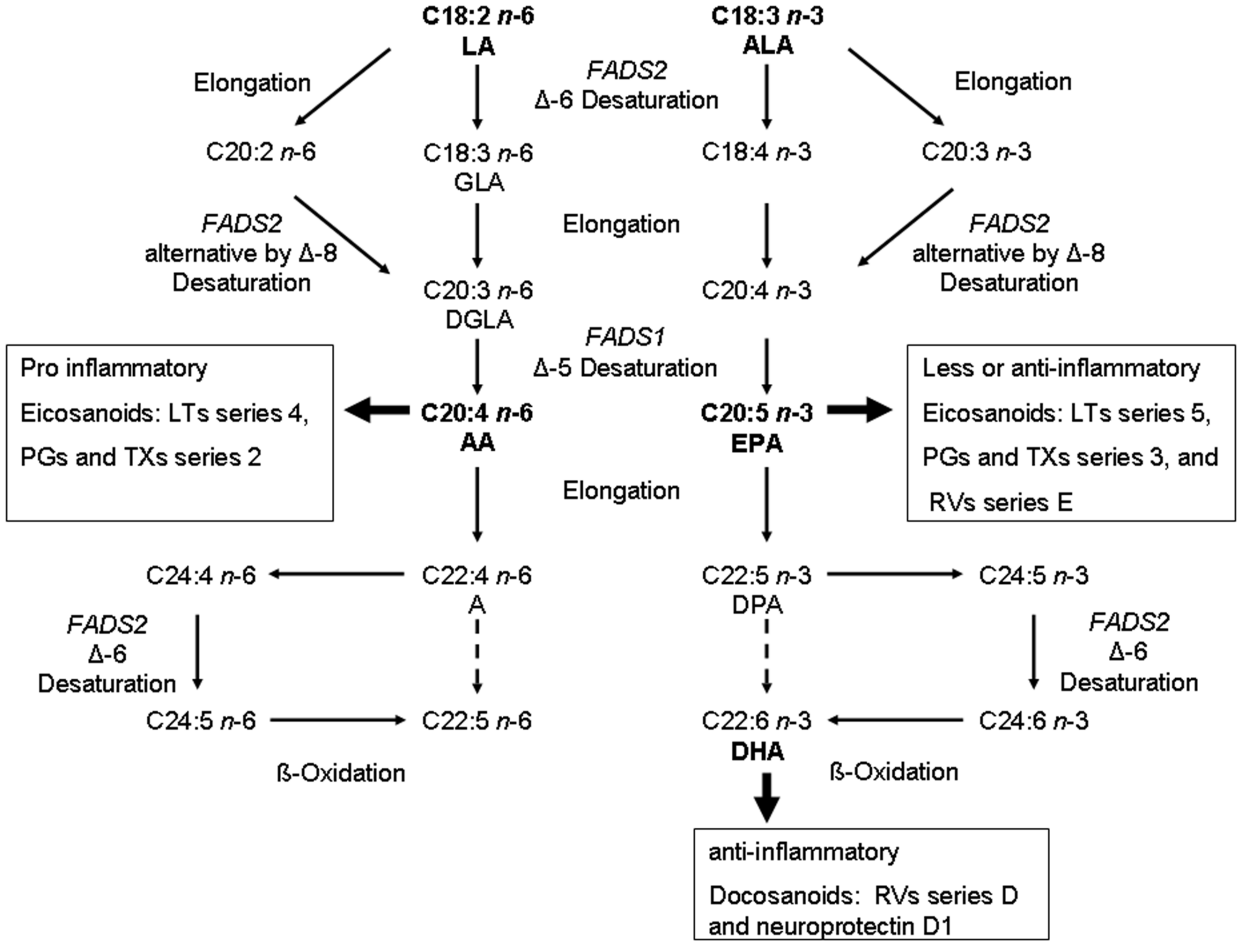
Metabolic pathways of n-6 and and n-3 fatty acids. Dashed arrows indicate pathways absent in human mammals. Figure is based on modified figures form Duchen et al. Glaser et al., and Park et al. [11], [12], [40]. LA = linoleic acid, GLA = gamma linolenic acid, DGLA = Dihomo-gamma-linolenic acid, AA = arachidonoc acid, A = adrenic acid, ALA = alpha-limolenic acid, EPA, = eicospentaenoic acid, DPA = docosapentaenoic acid, DHA = docosahexaenoic acid; LTs = leukotrienes, PGs = prostaglandins, TXs = thromboxanes, RVs = Resolvins.

Long chain polyunsaturated fatty acids (LC-PUFA) can influence inflammatory responses, as they are precursors of eicosanoids and docosanoids [16]. The link between PUFA and inflammatory processes are the eicosanoids with arachidonic acid (AA) as their main precursor. Eicosanoids derived from AA (e.g. leukotrienes LTB4, prostaglandins PGE2, PGI2 or thromboxanes TXA2) have mainly pro-inflammatory effects [16]. According to findings of several studies a defect in enzyme activity of Δ-6 desaturase, encoded by the *FADS2* gene, leads to enhanced blood levels of the n-6 and n-3 parent fatty acids linoleic (LA) and alpha-linolenic acid (ALA), respectively, and decreased levels of AA and eicosapentaenoic acid (EPA), whereas docosahexaenoic acid (DHA) levels are not influenced [5], [6]. Schaeffer et al. showed that the variability of serum fatty acid levels explained by genetic variants in the *FADS1 FADS2* gene cluster is highest for AA with 28% [6]. Thus, variants in the *FADS1 FADS2* gene cluster may be indirectly associated with inflammatory processes via their influence on endogenous LC-PUFA production, particularly AA production. **Figure 1** show these metabolic pathways of n-6 and n-3 fatty acids and pathways of production of pro-inflammatory and less inflammatory eicosanoids and anti-inflammatory docosanoids schematically [12].

No study has empirically confirmed the genetic link between the variants in the *FADS1 FADS2* gene cluster with polyunsaturated fatty acids (PUFA) in children. Schaeffer et al found an association of rarer *FADS* haplotypes with a reduced eczema risk in adults [6], but so far, no study has been published relating both genotypes *and* fatty acids to eczema development in children. Therefore, we analyzed this genetic association in a children cohort study pooled from two population based birth cohorts and investigate the association between variants in the *FADS* gene cluster, blood fatty acid composition and eczema up to the age of two years.

## Methods

### Ethics statement

Approval by the respective local Ethics Committees (Maastricht University/University Hospital of Maastricht, Bavarian General Medical Council) and written informed consent from participants' families (parents) were obtained in both KOALA and LISA studies.

### Study design and population

The KOALA Birth Cohort Study (“*Kind, Ouders en gezondheid: Aandacht voor Leefstijl en Aanleg*”) is a prospective cohort study of 2834 mother-infant pairs in the Netherlands. Aim of the KOALA study was to investigate factors that influence the clinical expression of atopic disease with a main focus on lifestyle factors (e.g. anthroposophy, dietary habits, breastfeeding, intestinal micro flora, and gene-environment-interactions). Enrollment started in October 2000. Details of the study design have been described elsewhere [17], [18].

The LISA study (“*Influences of Lifestyle related Factors on the Immune System and the Development of Allergies in Childhood*”) is an ongoing population-based birth cohort study of unselected 3097 newborns. The study was designed to assess ‘Influences of Lifestyle related Factors on the Immune System and the Development of Allergies in Childhood’. Between November 1997 and January 1999, n = 3097 healthy full-term newborns (gestational age≥37 weeks) were recruited from 14 obstetrical clinics in Munich (n = 1467), Leipzig (n = 976), Wesel (n = 306), and Bad Honnef (n = 348). Details on study design are published elsewhere [19], [20].

The studied analysis population with information on genotypes, fatty acids and eczema at 2 years of age comprised 546 children in the KOALA birth cohort study and 333 in the LISA birth cohort study.

For details on both studies **(see Supporting Information Appendix S1)**.

### Fatty acid analysis

In the KOALA study blood was collected in EDTA-tubes during a home visit to the child around age 2 years, by trained nurses according to a standardized protocol. The EDTA-plasma was used for the analysis of the fatty acid status and was, after centrifugation, stored in cryovials at −80°C. The EDTA-plasma was deproteinated and the precipitate was removed by centrifugation. Then the sample was applied to an aminopropyl-solid-phase column to selectively elute the phospholipid fraction [21], [22].

In the LISA study venous blood samples were collected in serum-separator-tubes. Samples were centrifuged and serum was frozen in plastic vials and stored at −80°C until analysis. The analysis of plasma-glycerophospholipids composition was performed by a sensitive and precise high-throughput method as described recently [23].

For details **(see Supporting Information Appendix S2)**.

### Genotyping

In KOALA, genomic DNA was extracted from buccal swabs using standard methods as described previously [24]. In LISA, genomic DNA was extracted from EDTA blood.

Five variants of the *FADS1 FADS2* gene cluster (rs174545, rs174546, rs174556, rs174561, rs3834458) were typed with MALDI-TOF-MS.

For details **(see Supporting Information Appendix S3)**.

### Determination of infants' IgE

In the KOALA study total and specific IgE were determined at age 1 (against hen's eggs, cow's milk, and peanuts) and 2 years (against eggs, cow's milk, peanut, birch pollen, grass pollen, cat, dog and house-dust mite) by blood sampling during home visits [25]–[29].

In the LISA study blood samples were collected during physical examination of the infant at age 2 years and analysed for total and specific IgE using the RAST-CAP-FEIA-system (Pharmacia, Freiburg, Germany) as previously described [30]. For details **(see Supporting Information Appendix S4)**.

### Definition of outcome variable parental reported eczema

In both the KOALA and LISA study the definition of “parental reported eczema” is based on the questionnaire-reported occurrence of “itchy rash that was coming and going” at any time within the first two years of life [18], [31].

For details **(see Supporting Information Appendix S5)**.

### Statistical analysis

Allele frequencies, Fisher's exact test of Hardy-Weinberg-Equilibrium (HWE), the linkage disequilibrium (LD) tests Lewontin's D' and pairwise-squared correlations r^2^ were calculated for the study population and for both studies separately.

Single SNP linear regression analyses for the relation between *FADS* variants and the nine continuous outcome variables (fatty acids) were conducted applying an additive coded model. *P*-Values were corrected for multiple testing by Bonferroni correction.

Logistic regression was applied to evaluate the effect of each single SNP and of each fatty acid separately on the dichotomous coded outcome “parental reported eczema during the first two years of life”. No corrections for multiple testing were applied in logistic regression.

All analyses were performed using the statistical software SAS, version 9.1.3 [32], [33], except calculation of linkage disequilibrium measures (JLIN) [34].

For details **(see Supporting Information Appendix S6)**.

## Results

Study characteristics for both study populations separately and combined are listed in **Table 1**.

**Table 1:** Characteristics of study population.

	KOALA-Study			LISA-Study			Total		
	n	mean or %	95%-CI	n	mean or %	95%-CI	n	mean or %	95%-CI
% boys	546	50.4	(46.2–54.6)	333	55.6	(50.2–60.9)	879	52.3	(49.0–55.6)
% high maternal education	543	58.9	(54.8–63.1)	330	66.7	(61.6–71.8)	873	61.9	(58.6–65.1)
% parental asthma or allergy	546	57.0	(52.8–61.1)	332	68.7	(63.7–73.7)	878	61.4	(58.2–64.6)
%maternal smoking during pregnancy	546	3.1	(1.7–4.6)	330	10.3	(7.0–13.6)	876	5.8	(4.3–7.4)
% exclusively breastfed for at least 3 months	538	50.0	(45.8–54.2)	331	71.6	(66.7–76.5)	869	58.2	(54.9–61.5)
fatty acids (mean % wt of total FA)									
Linoleic (LA)	546	21.37	(21.14–21.60)	333	22.22	(21.92–22.52)	879	21.69	(21.51–21.88)
γ-Linolenic (GLA)	542	0.07	(0.07–0.08)	333	0.11	(0.10–0.11)	875	0.09	(0.08–0.09)
ln (GLA)^a^	542	−2.93	(−3.00–−2.85)	333	−2.33	(−2.37–−2.29)	875	−2.70	(−2.75–−2.65)
Dihomo-γ-linolenic (DGLA)	546	2.82	(2.77–2.87)	333	2.91	(2.84–2.97)	879	2.85	(2.81–2.89)
Arachidonic (AA)	546	9.06	(8.95–9.16)	333	9.33	(9.19–9.47)	879	9.16	(9.08–9.25)
Adrenic (A)	545	0.47	(0.46–0.48)	333	0.40	(0.39–0.41)	878	0.44	(0.44–0.45)
α-Linolenic (ALA)	544	0.21	(0.21–0.22)	333	0.24	(0.23–0.25)	877	0.22	(0.22–0.23)
ln (ALA)^a^	544	−1.61	(−1.64–−1.58)	333	−1.49	(−1.52–−1.45)	877	−1.56	(−1.59–−1.54)
Eicosapentaenoic (EPA)	546	1.00	(0.98–1.03)	333	0.55	(0.52–0.58)	879	0.83	(0.81–0.86)
ln (EPA)^a^	546	−0.05	(−0.09–−0.02)	333	−0.68	(−0.72–−0.63)	879	−0.29	(−0.32–−0.26)
n-3 Docosapentaenoic (DPA)	546	0.92	(0.91–0.94)	333	0.97	(0.95–0.99)	879	0.94	(0.93–0.95)
Docosahexaenoic (DHA)	546	2.67	(2.61–2.74)	333	3.07	(2.99–3.15)	879	2.82	(2.77–2.88)
ln (DHA)^a^	546	0.95	(0.93–0.97)	333	1.09	(1.07–1.12)	879	1.00	(0.99–1.02)
% parental reported eczema of child in first 2 years of life	542	30.6	(26.7–34.5)	333	14.1	(10.4–17.9)	875	24.3	(21.5–27.2)
IgE at 2 years of life									
mean total IgE (IU/ml)	544	59.3	(34.9–83.7)	327	56.3	(44.3–68.3)	871	58.2	(42.3–74.0)
% specifc IgE (egg)	544	5.5	(3.6–7.4)	333	4.8	(2.5–7.1)	877	5.2	(3.8–6.7)
% specifc IgE (milk)	544	16.9	(13.8–20.1)	333	4.2	(2.0–6.4)	877	12.1	(9.9–14.2)
% specifc IgE (peanut)	544	5.0	(3.1–6.8)	333	1.2	(0.0–2.4)	877	3.5	(2.3–4.8)
% specifc IgE (birch)^b^	535	0.4	(−0.1–0.9)	-	-	-	-	-	-
% specifc IgE (gras)^b^	538	1.9	(0.7–3.0)	-	-	-	-	-	-
% specifc IgE (cat)	543	2.8	(1.4–4.1)	333	0.6	(−0.2–1.4)	876	1.9	(1.0–2.9)
% specifc IgE (dog)^c^	543	2.6	(1.2–3.9)	-	-	-	-	-	-
% specifc IgE (dust mite)	544	6.8	(4.7–8.9)	333	0.6	(−0.2–1.4)	877	4.7	(3.3–6.1)

a Mean levels of GLA, ALA, EPA and DHA are naturally logged means to account for the severely skewed distributions of these fatty acids; see methods section.

b In the LISA study % specific IgE for sensitization to birch and gras was not determined separately but for a mix of grass, trees and herbs only (0.9% (95%-CI −0.1–1.9)) at age 2.

c In the LISA study % specific IgE for sensitization to dog was not determined at age 2.

There are some differences between both studies in the percentages of boys, high maternal education, family history of asthma or allergy, proportion of maternal smoking during pregnancy and exclusive breastfeeding. Mean percentage contributions of fatty acids to plasma phospholipids in the KOALA cohort and to plasma glycerophospholipids in the LISA cohort are similar, except for an almost twofold higher percentage value of EPA in the KOALA study, which might be related to the different population studied with a potentially higher fish consumption in the Netherlands, as well as the different analytical method used.

Information regarding position, possible functional region and genotyping frequencies for the five analyzed SNPs of the *FADS1 FADS2* gene cluster **(see Supporting Information Table S1)**. Minimum *P*-value of Fisher's exact test for violation of Hardy-Weinberg-Equilibrium (HWE) for any of the five SNPs was 0.48 (rs174546) in the whole study population and 0.62 and 0.61 for the KOALA and LISA study populations separately.

**Figure 2:**
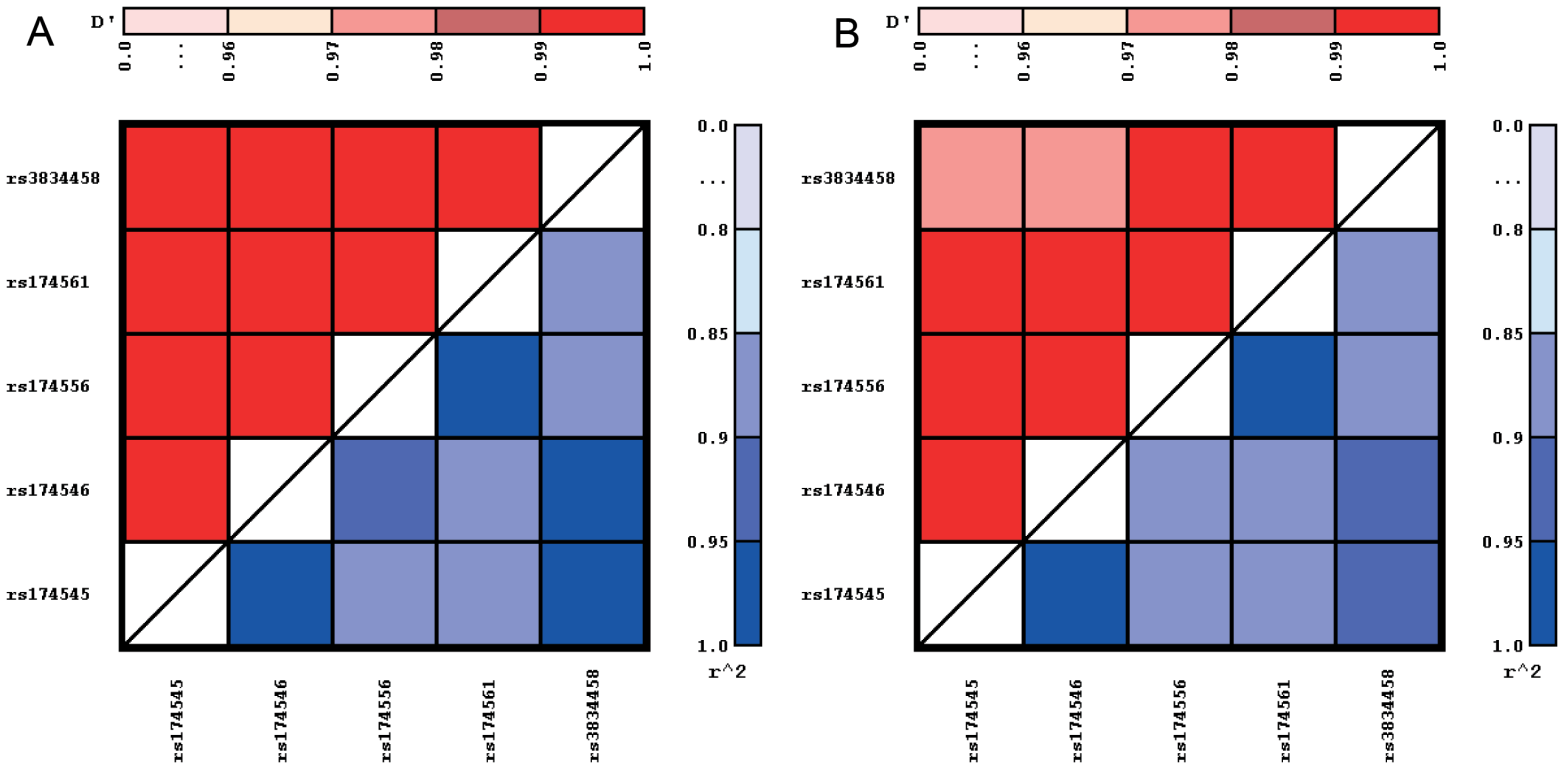
Pairwise linkage disequilibrium measured by Lewontin's D' and r^2^ for the common five single nucleotide polymorphisms (SNP) in the KOALA (Panel A) and the LISA (Panel B) birth cohort studies.

Lewontin's D' and pairwise-squared correlations r^2^ for the KOALA study and for the LISA study are depicted in **Figure 2**. In the KOALA study Lewontin's D' ranged between 0.99 and 1.0 and the pairwise-squared correlations r^2^ ranged between 0.89 and 0.99. For the LISA study D' ranged between 0.97 and 1.0 and r^2^ between 0.87 and 1.0. Both measures confirm that all five SNPs are in high linkage disequilibrium (LD).

### Single SNP associations with fatty acids

Mean levels and standard deviations for each fatty acid by genotype of each SNP (rs174545, rs174546, rs174556, rs174561, rs3834458) for the combined study population and for the KOALA and LISA studies separately are listed in in the Online Repository **(see Supporting Information Table S2, Table S3 and Table S4)**. Note that mean levels of GLA, ALA, EPA and DHA are naturally logged means to account for the severely skewed distributions of these fatty acids.

Single SNP regression analyses of each additive coded SNP (0/1/2) with each fatty acid in the combined study population of the KOALA and LISA studies at age 2 years revealed that all SNPs are highly significantly associated with all fatty acids except for ALA and EPA (natural log scale, **Table 2**). This is true even though all *P*-values have been conservatively corrected for multiple testing according to Bonferroni's method. With respect to ALA, corrected significance at the 5% level was only reached for rs174545 and rs3834458 but not for the other SNPs. Regarding EPA, regression coefficients were in the same direction and the same order of magnitude as for DPA and DHA, but corrected *P*-values were only between 0.05 and 0.09. Separate analyses for both study populations revealed that in the LISA study associations of the SNPs were significant only with LA, GLA, DGLA, AA and EPA after correction for multiple testing **(see Supporting Information Table S5 and Table S6)**. In the KOALA study all SNPs showed a significant association with all n-6 and n-3 PUFAs except EPA. However, regression coefficients of SNPs were in the same direction for all PUFAs so the lack of significance regarding n-3 PUFA in the LISA study may just reflect a loss of power due to the smaller sample size of the LISA study compared to that of the KOALA study.

**Table 2:** Associations of the five analyzed variants in the *FADS1 FADS 2* gene region with fatty acids.

	Fatty acid	LA	GLA	DGLA	AA	A	ALA	EPA	DPA	DHA
SNP		C18_2n_6	ln(C18_3n_6)	C20_3n_6	C20_4n_6	C22_4n_6	ln(C18_3n_3)	ln(C20_5n_3)	C22_5n_3	ln(C22_6n_3)
rs174545										
	*P-value (corrected)*	4.1E-10	1.3E-04	6.5E-13	5.4E-38	0.003	0.039	0.071	1.7E-05	3.2E-04
	ß-coefficient	0.964	−0.191	0.224	−0.818	−0.021	0.057	−0.080	−0.046	−0.059
	R^2^ (explained var.)	5.3%	2.5%	6.7%	18.3%	1.8%	2.5%	1.2%	3.0%	3.3%
rs174546										
	*P-value (corrected)*	3.7E-10	7.9E-05	1.4E-12	2.9E-39	0.002	0.066	0.066	5.9E-06	1.5E-04
	ß-coefficient	0.962	−0.194	0.221	−0.828	−0.022	0.055	−0.080	−0.048	−0.061
	R^2^ (explained var.)	5.3%	2.6%	6.5%	18.8%	2.0%	2.6%	1.2%	3.2%	2.5%
rs174556										
	*P-value (corrected)*	1.1E-09	0.002	2.3E-13	3.9E-37	0.009	0.218	0.054	1.4E-06	6.3E-05
	ß-coefficient	0.975	−0.170	0.235	−0.836	−0.021	0.050	−0.085	−0.052	−0.065
	R^2^ (explained var.)	5.1%	1.9%	6.7%	17.9%	1.6%	1.9%	1.2%	3.5%	2.7%
rs174561										
	*P-value (corrected)*	1.2E-09	0.002	5.0E-13	2.1E-37	0.005	0.384	0.051	9.8E-07	6.3E-05
	ß-coefficient	0.971	−0.174	0.232	−0.836	−0.021	0.047	−0.085	−0.053	−0.065
	R^2^ (explained var.)	5.0%	2.0%	6.7%	18.0%	1.7%	2.0%	1.2%	3.6%	2.7%
rs3834458										
	*P-value (corrected)*	3.9E-10	1.1E-04	3.8E-14	5.7E-38	0.005	0.018	0.092	5.9E-06	6.0E-05
	ß-coefficient	0.970	−0.193	0.236	−0.823	−0.019	0.061	−0.079	−0.049	−0.064
	R^2^ (explained var.)	5.3%	2.6%	7.3%	18.3%	1.7%	2.6%	1.1%	3.2%	2.6%

Note: Corrected *P*-values to account for multiple testing are obtained by dividing not corrected *P*-values by 45 (5 single SNPanalyses of 9 outcomes); ß-coeffcient is the respective regression estimate of the additive coded SNP (0/1/2) for the respective outcome (PUFA) in a simple regression model.

### Associations of single SNPs with parental reported eczema

Of all analyzed indicator coded SNPs of the *FADS1 FADS2* gene cluster none showed a statistically significant association with the dichotomous outcome parental reported eczema in the first 2 years of life (**Table 3**) in both unadjusted and adjusted analyses (see methods). Odds ratios (OR) for carriers of one or two minor alleles in comparison to non-carriers range from 1.3 to 1.5 in unadjusted, and 1.3 to 1.4 in adjusted analyses, respectively. However, 95%-confidence limits always include the null-effect of an OR of 1.0. Moreover, tests for multiplicative allelic trend (genotypes coded 0, 1, 2) also showed that none was significant at the 5% level with, even though no correction for multiple testing was applied.

**Table 3:** Association of *FADS1 FADS 2* variants with parental reported eczema.

SNP		n	OR	95%-CI	*P-value Trend*	n	OR	95%-CI	*P-value Trend*
		unadjusted				adjusted			
rs174545	C/C	399	1.00	-	0.07	392	1.00	-	0.13
	C/G	364	1.32	0.95–1.85		357	1.32	0.94–1.87	
	G/G	93	1.43	0.86–2.39		92	1.34	0.79–2.28	
rs174546	C/C	401	1.00	-	0.09	394	1.00	-	0.16
	C/T	363	1.30	0.93–1.81		356	1.30	0.92–1.83	
	T/T	94	1.40	0.84–2.33		93	1.30	0.77–2.21	
rs174556	C/C	424	1.00	-	0.05	417	1.00	-	0.12
	C/T	358	1.34	0.96–1.86		351	1.33	0.95–1.87	
	T/T	76	1.49	0.86–2.58		75	1.33	0.75–2.35	
rs174561	T/T	427	1.00	-	0.07	420	1.00	-	0.15
	T/C	356	1.31	0.94–1.82		349	1.31	0.93–1.84	
	C/C	77	1.46	0.84–2.51		76	1.30	0.74–2.29	
rs3834458	T/T	396	1.00	-	0.06	389	1.00	-	0.10
	T/Z	370	1.28	0.92–1.79		363	1.28	0.91–1.81	
	Z/Z	91	1.54	0.93–2.57		90	1.42	0.84–2.42	

Note: Odds ratios (OR) of indicator coded SNPs (reference is homozygous major alleles genotype) on eczema are estimated by logistic regression. Adjustment comprises study cohort (indicator coded dummy variables: KOALA study conventional, KOALA study alternative recruitment group vs. LISA study as reference), sex, maternal education, maternal smoking during pregnancy and exclusive breastfeeding for at least 3 months.

Separate analyses for both study populations revealed that in the LISA-study all SNPs of the *FADS1 FADS2* gene cluster showed significant associations with the dichotomous outcome parental reported eczema in the first 2 years of life in both unadjusted and adjusted analyses, with ORs of about 2 and 4 for heterzygous and homozygous minor allele carriers, which is in good agreement with multiplicative increase of risks (confirmed by *P*-values for multiplicative trend between 0.003 and 0.005 without correction for multiple testing **(see Supporting Information Table S7)**. By contrast, no associations were found within the KOALA study, where all ORs were between 0.9 and 1.2, whereas upper boundaries of the 95% confidence intervals were below 2.0.

No significant association of any analyzed SNP of the *FADS* gene with total IgE level or specific IgE at the age of one or two years of the child could be established, neither in the KOALA nor in the LISA study (results not presented).

### Associations of fatty acids with parental reported eczema

No single PUFA was significantly associated with parental reported eczema of the child in the first 2 years of life after adjustment (see methods), neither in analyses of the combined study population (**Table 4**), nor in separate analyses for the KOALA and LISA studies **(see Supporting Information Table S8)**.

**Table 4:** Association of fatty acids with parental reported eczema.

Fatty Acid		n	OR	95%-CI	*P-value*	n	OR	95%-CI	*P-value*
		unadjusted				adjusted			
LA	C18_2n_6	875	0.99	0.94–1.05	0.85	860	1.03	0.97–1.09	0.39
GLA	ln(C18_3n_6)	871	0.00	0.00–0.08	0.00	856	0.88	0.72–1.07	0.19
DGLA	C20_3n_6	875	0.92	0.71–1.21	0.57	860	0.95	0.72–1.25	0.71
AA	C20_4n_6	875	1.00	0.89–1.13	0.97	860	1.02	0.90–1.15	0.80
A	C22_4n_6	874	3.41	0.82–14.16	0.09	859	0.85	0.17–4.15	0.84
ALA	ln(C18_3n_3)	873	0.79	0.50–1.25	0.32	858	0.99	0.61–1.61	0.97
EPA	ln(C20_5n_3)	875	1.53	1.09–2.15	0.01	860	0.82	0.55–1.21	0.31
DPA	C22_5n_3	875	0.46	0.19–1.11	0.08	860	0.60	0.24–1.50	0.27
DHA	ln(C22_6n_3)	875	0.87	0.48–1.59	0.66	860	1.53	0.80–2.95	0.20

Note: Odds ratios (OR) of measured PUFA on eczema are estimated by logistic regression. Adjustment comprises study cohort (indicator coded dummy variables: KOALA study conventional, KOALA study alternative recruitment group vs. LISA study as reference), sex, maternal education, maternal smoking during pregnancy and exclusive breastfeeding for at least 3 months.

## Discussion

This is the first study presenting associations between the genetic variants in the *FADS1 FADS2* gene cluster with fatty acid composition in a population based sample of children.

We found that all five analyzed variants of the *FADS1 FADS2* gene cluster are associated with polyunsaturated fatty acids LA, GLA, DGLA, AA, A, ALA, EPA, DPA and DHA, in particular with AA. Except for the n-3 fatty acids ALA and EPA these associations are highly significant even after conservative Bonferroni correction for multiple testing. For most PUFAs this is in line with previous reports showing these associations in adult populations [4]–[8]. However, in contrast to previous studies in adults, all SNPs in the present study in children are also highly significantly associated with DHA in one of the study populations (KOALA study). In the same population, Moltó et al found a relation between DHA in blood of the KOALA mothers during pregnancy and the mothers' *FADS* variants [35].

Our study confirms previous reports that carriers of the minor alleles showed higher levels of n-6 precursor fatty acids LA, DGLA and n-3 precursor fatty acids ALA and decreased product levels of the n-6 fatty acids GLA, AA, A and n-3 fatty acids EPA, DPA and DHA. Thus carriers of the minor alleles show enhanced desaturase substrate levels (substrate accumulation) and decreased desaturase product levels indicative of a lower desaturase activity, in agreement with previous studies [5], [6]. Thus, our results confirm that the *FADS1 FADS2* gene cluster modulates the PUFA metabolism and demonstrate that this is also the case in children at the age of 2 years. A functional basis for such a regulation was recently shown by Lattka et al. in a study investigating the *FADS2* variants rs3834458 and rs968567 [36]. According to this study both SNPs are potential promoter polymorphisms and located in a region important for transcription regulation. The minor alleles of SNP rs968567, but not rs3834458 (studied also in our study), showed a statistical significant increased effect on promoter activity and binding activity to two protein complexes activating the transcription factor ELK1 in that study. However, one of the rare other functional studies on *FADS2* found a decreased promoter activity for rs3834458, suggesting the need for many more functional studies [37].

A previous study in adults found protective associations of carriers of minor alleles of the *FADS1 FADS2* gene cluster with allergic rhinitis and atopic eczema [6]. In our analyses we could confirm such an association between *FADS* variants and eczema only for the German study population, but clearly not within the larger sample of children of the Netherlands. Whether these inconsistent results suggest that in children less common variants of the *FADS1 FADS2* gene cluster are actually related to the development of eczema within the first 2 years of life requires further investigations with further independent study populations.

In contrast to some previous studies summarized in several reviews [9]–[11], we found no evidence for a direct link between the analyzed blood PUFA levels and eczema. This was true for analyses based on the combined study population as well as for separate analyses of the KOALA and LISA cohorts. Apparently, the development of disease cannot be simplified to one underlying pathway on fatty acid metabolism. On the other hand, larger sample sizes and analyses in further study populations may be necessary to draw any final conclusion on the role of fatty acid composition in blood and eczema. Also, studies of gene-diet interactions may be needed to resolve these inconsistencies, since populations vary widely in the intake n-3 PUFAs from fish products.

### Strengths and Limitations

With data on variants of the *FADS1 FADS2* gene cluster, PUFA and information on eczema status within the first 2 years of life for more than 800 children this is a large study despite the potential lack of power to detect small effects regarding eczema.

Genotyping for both study populations (KOALA, LISA) were done in the same lab at the Helmholtz Center Munich.

Fatty acids were derived from phospholipids in the KOALA study and from serum glycerophospholipids in the LISA study and analyzed at two different labs by somewhat different methods, but we do not think that this compromised our results. Both labs (Laboratory for Health Protection Research, National Institute for Public Health and the Environment, Bilthoven, the Netherlands and Division of Metabolic Diseases and Nutritional Medicine, Dr. von Hauner Children's Hospital, Ludwigs-Maximilians-University of Munich) have a long lasting experience in fatty acid analysis. Moreover, Glaser et al. have recently shown that fatty acid analysis of serum glycerophospholipids by high throughput analysis results in only minor deviations in values of fatty acids compared to analysis of serum phospholipid fatty acids [23]. Indeed, the fatty acid composition data was quite similar for both study populations, except for EPA, which might reflect different dietary habits with higher consumption levels of fish, which is the prime dietary source of EPA, in the Netherlands than in Bavaria (cf. Table 1).

We used a similar definition of the outcome “parental reported eczema of child within the first 2 years of life”, but slightly different wording of the questions and slightly different timing of questionnaires, covering the entire first two years of life in the two cohorts. Despite these similarities we found more than double the percentage in parental reported eczema in the children of the KOALA study (30.6%) than in the sample of the German LISA study (14.1%), even though in KOALA parents the prevalence of asthma and allergies was lower (57.0%) than in LISA parents (68.7%). This raises the question of whether lifestyle differences are responsible for the marked difference in % eczema in children in the two cohorts. There are two additional elements which further press this question: First, the *FADS1* and *FADS2* polymorphism prevalence is remarkably similar in the two populations, suggesting similar genetic background in the two populations. Second, in the LISA study, 71% of children are exclusively breastfed vs. only 50% in the KOALA study. To compare, the reported prevalence on eczema defined by symptoms in young children in two other birth cohort studies are 25% in children of age 4 years in the Netherlands and 16% for the first 2 years of life years in Germany [38], [39]. However, we do not think that any differences in eczema between the LISA and KOALA study affected the results to an appreciable extent, since we conducted, in addition to unadjusted analyses, also analyses adjusted for study cohort. The higher prevalence of eczema in KOALA could point to a higher proportion of non-atopic eczema, and if the *FADS* effect would be confined to atopic (IgE-mediated) eczema, it could have been diluted by non-atopic cases in the KOALA study. However, the absence of a *FADS* effect on total and specific IgE in both cohorts makes this explanation unlikely.

At present we have no convincing explanation, why we found a significant association between variants of the *FADS* gene and eczema only in the German LISA study, but not in the Netherland's KOALA study. Since Moltó et al found that the DHA deficit in the homozygous minor allele carriers could be overcome by intake of fish at the recommended level of 2 portions a week [35] we speculate that the difference between LISA and KOALA may reflect a lower intake of n-3 long chain PUFAs in LISA, as a consequence of which the genetic effect is manifest in LISA but not in KOALA. Based on this, we recommend that further studies are done in populations with low n-3 PUFA intake.

*In conclusion*, this is the first study that confirms in children of age 2 years the previously found associations of genetic variants in the *FADS1 FADS2* gene cluster with fatty acid composition in serum phospholipids or glycerophospholipids and that also analyzed the potential influence of *FADS1 FADS2* genotypes and PUFAs to eczema.

Variants of the *FADS1 FADS2* gene cluster clearly do regulate the metabolism of PUFA in young children. Inconsistent results were found for a link between these genetic variants with eczema. In the German LISA study all SNPs were significantly associated with eczema. In the Netherland's KOALA study this was clearly not the case. In both study populations PUFA was not associated with eczema. Thus the hypothesis of an inflammatory link between PUFA and eczema by the metabolic pathway of LC-PUFAs as precursors for inflammatory prostaglandins and leukotrienes could not be confirmed by these data. This would suggest either that this pathway is not or only marginally involved in eczema development, that other risk factors for early childhood eczema may be more important, or that eczema may have heterogeneous etiology with only a small segment of the population susceptible for effects of endogenous fatty acids metabolism or gene-diet-interaction; or a combination of these arguments.

## Supporting Information

Appendix S1 Study design and population
(0.04 MB DOC)

Appendix S2 Fatty acid analysis
(0.04 MB DOC)

Appendix S3 Genotyping
(0.04 MB DOC)

Appendix S4 Determination of infants' IgE
(0.04 MB DOC)

Appendix S5 Definition of outcome variable parental reported eczema
(0.04 MB DOC)

Appendix S6 Statistical analysis
(0.04 MB DOC)

Table S1 Characteristics of the five analyzed variants in the FADS1 FADS 2 gene region
(0.37 MB DOC)

Table S2 Mean levels of fatty acids (PUFA) by genotype of five variants in the FADS1 FADS 2 gene region (KOALA & LISA study combined)
(0.39 MB DOC)

Table S3 Mean levels of fatty acids (PUFA) by genotype of five variants in the FADS1 FADS 2 gene region in the KOALA study
(0.39 MB DOC)

Table S4 Mean levels of fatty acids (PUFA) by genotype of five variants in the FADS1 FADS 2 gene region in the LISA study
(0.39 MB DOC)

Table S5 Associations of the five analyzed variants in the FADS1 FADS 2 gene region with fatty acids in the KOALA study
(0.39 MB DOC)

Table S6 Associations of the five analyzed variants in the FADS1 FADS 2 gene region with fatty acids in the LISA study
(0.39 MB DOC)

Table S7 Association of FADS1 FADS 2 variants with parental reported eczema
(0.38 MB DOC)

Table S8 Association of fatty acids with parental reported eczema
(0.38 MB DOC)
