# Supplementary material for: Variants of the FADS1 FADS2 Gene Cluster, Blood Levels of Polyunsaturated Fatty Acids and Eczema in Children within the First 2 Years of Life
Source: PLoS One. 2010 Oct 11;5(10):e13261. doi: 10.1371/journal.pone.0013261 (PMC2952585; doi:10.1371/journal.pone.0013261)
Supplement: Appendix S1 — Study design and population (0.04 MB DOC) [file pone.0013261.s001.doc]

**Supporting Information Appendix S1 Study design and population**

The KOALA Birth Cohort Study (“*Kind, Ouders en gezondheid: Aandacht voor Leefstijl en Aanleg”*) is a prospective cohort study of 2834 mother-infant pairs in the Netherlands. Aim of the KOALA study was to investigate factors that influence the clinical expression of atopic disease with a main focus on lifestyle factors (e.g. anthroposophy, dietary habits, breastfeeding, intestinal micro flora, and gene-environment interactions). Enrollment started in October 2000. Details of the study design have been described elsewhere.^S1,S2^

Inclusion in the KOALA cohort was based on availability of the baseline questionnaire at 34 weeks of pregnancy (2343 pregnancies in the conventional and 491 in the alternative lifestyle recruitment group, total 2834 pregnancies). There were 20 twin pregnancies, from whom only the first born child was included. After exclusions (2 intrauterine and 3 neonatal deaths, 7 infants with Down’s syndrome and 7 other congenital syndromes) 2815 children remained (2329 in the conventional and 586 in the alternative recruitment group). DNA was collected from buccal swabs by the parents in 1640 children: 1303 (56%) in the conventional and 337 (58%) in the alternative recruitment group. Follow-up questionnaires were filled out by parents of 1624 (99%) of the children at age 2 years (1290 and 334 in the respective recruitment groups; 824 boys and 800 girls) and blood samples were collected for fatty acids determinations in 546 children at age 2 years (382 and 164 in the respective recruitment groups; 275 boys and 271 girls), and in all of them genotyping data for the five single nucleotide polymorphisms (SNPs) of the *FADS1 FADS2* gene cluster and fatty acid data were available.

The LISA study (“*Influences of Lifestyle related Factors on the Immune System and the Development of Allergies in Childhood”*) is an ongoing population-based birth cohort study of unselected newborns. The study was designed to assess ‘Influences of Lifestyle related Factors on the Immune System and the Development of Allergies in Childhood’. Between November 1997 and January 1999, n=3097 healthy full-term newborns (gestational age ≥37 weeks) were recruited from 14 obstetrical clinics in Munich (n=1467), Leipzig (n=976), Wesel (n=306), and Bad Honnef (n=348). Children were followed up at 0, 0.5, 1, 1.5 and 2 years after birth by questionnaire. Blood sampling was performed at birth and at 2 years of age. Details on study design are published elsewhere.^S3,S4^

For the present analyses, only data from the 2-year follow-up of study center Munich were included (n=1331), and blood samples were taken in 1062 children. Genotyping data on five SNPs of the *FADS1 FADS2* gene cluster were available for 1037 children. There were seven twin pregnancies, from whom only the first born child was included (n=1030). Fatty acid data were available for 737 children (412 boys, 325 girls). Both genotyping data and fatty acid data were available only for 333 children.

Approval by the respective local Ethics Committees (Maastricht University/ University Hospital of Maastricht, Bavarian General Medical Council) and written informed consent from participants’ families were obtained in both KOALA and LISA studies.

S1 Kummeling I, Thijs C, Penders J, Snijders BE, Stelma F, et al. (2005) Etiology of atopy in infancy: the KOALA Birth Cohort Study. Pediatr Allergy Immunol; 16:679-84.

S2 Snijders BE, Thijs C, van Ree R, van den Brandt PA (2008) Age at first introduction of cow milk products and other food products in relation to infant atopic manifestations in the first 2 years of life: the KOALA Birth Cohort Study. Pediatrics; 122:e115-e122.

S3 Heinrich J, Bolte G, Holscher B, Douwes J, Lehmann I, et al. (2002) Allergens and endotoxin on mothers' mattresses and total immunoglobulin E in cord blood of neonates. Eur Respir J; 20:617-23.

S4 Sausenthaler S, Koletzko S, Schaaf B, Lehmann I, Borte M, et al. (2007) Maternal diet during pregnancy in relation to eczema and allergic sensitization in the offspring at 2 y of age. Am J Clin Nutr; 85:530-7.
