# Supplementary material for: Variants of the FADS1 FADS2 Gene Cluster, Blood Levels of Polyunsaturated Fatty Acids and Eczema in Children within the First 2 Years of Life
Source: PLoS One. 2010 Oct 11;5(10):e13261. doi: 10.1371/journal.pone.0013261 (PMC2952585; doi:10.1371/journal.pone.0013261)
Supplement: Appendix S2 — Fatty acid analysis (0.04 MB DOC) [file pone.0013261.s002.doc]

Supporting Information Appendix S2 Fatty acid analysis

In the KOALA study blood was collected in EDTA-tubes during a home visit to the child around age 2 years, by trained nurses according to a standardized protocol. The EDTA-plasma was used for the analysis of the fatty acid status and was, after centrifugation, stored in cryovials at -80ºC. The EDTA-plasma was deproteinated and the precipitate was removed by centrifugation. Then the sample was applied to an aminopropyl solid-phase column to selectively elute the phospholipid fraction.^S1,S2^ The phospholipids in the sample were then hydrolyzed and methylated simultaneously. After hexane extraction, the FAME-sample was analyzed using a GC-3900 gas chromatograph (Varian Inc) equipped with a CP 8400 auto injector. Galaxie software was used for quantization and identification of peaks. Results were expressed as weight percentage (% wt/wt) of all phospholipid FA detected with a chain length between 14 and 22 carbon atoms.

In the LISA study venous blood samples were collected in serum separator tubes. Samples were centrifuged and serum was frozen in plastic vials and stored at -80 °C until analysis. The analysis of plasma glycerophospholipids (GP) composition was performed by a sensitive and precise high-throughput method as described recently.^S3^ In brief, 100 µl of serum or plasma, 100 µl of internal standard (1,2-dipentadecanoyl-sn-glycero-3-phosphocholine dissolved in methanol) and 0.6 ml methanol (precooled to 5 °C) were combined in glass tubes and shaken for 30 s. Samples were centrifuged and the supernatant was transferred into another glass tube. Sodium methoxide solution (25 µl) was added, tubes were shaken and synthesis of methyl esters proceeded at room temperature. The reaction was stopped after 3 minutes by adding 75 µl methanolic HCl. FAME were extracted twice by adding 300 µl hexane at a time. Extracts were combined and dried under nitrogen flow at room temperature and taken up in 50 µl hexane (containing 2 g/l BHT) for gas chromatographic (GC) analysis.

Individual FAMEs were quantified by GC with flame ionization detection and identified by comparison with authentic standards. Response relative to pentadecanoic acid methyl ester (internal standard) was determined using GLC-85 (Nu-Check Prep, Inc., Elysian, MN, USA) as an external standard. EZChrom Elite version 3.1.7 (Agilent, Waldbronn, Germany) was used for peak integration. Results were expressed as weight percentage (% wt/wt) of all glycerophospholipid FA detected with a chain length between 14 and 24 carbon atoms.

For quality assurance, aliquots of a control serum were stored in plastic vials at -80 °C. One control serum aliquot was analyzed with every sample batch (serum samples which were simultaneously processed).

Note that a comparison between conventional methods for analyzing fatty acid (FA) composition in phospholipids (as conducted in the KOALA study) and the high-throughput method of analyzing glycerophospholipids (as performed in the LISA study) showed very good agreement between these methods. Coefficient of Variation for FA contributing more than 1% to total FA for the new method was less than 4% and correlations between the conventional and the new method were above 0.9.^S3^

S1 Agren JJ, Julkunen A, Penttila I (1992) Rapid separation of serum lipids for fatty acid analysis by a single aminopropyl column. J Lipid Res; 33:1871-6.

S2 Folch J, Lees M, Sloane Stanley GH (1957) A simple method for the isolation and purification of total lipides from animal tissues. J Biol Chem; 226:497-509.

S3 Glaser C, Demmelmair H, Koletzko B (2010) High-throughput analysis of fatty acid composition of plasma glycerophospholipids. J Lipid Res; 51:216-21.
