# Supplementary material for: Variants of the FADS1 FADS2 Gene Cluster, Blood Levels of Polyunsaturated Fatty Acids and Eczema in Children within the First 2 Years of Life
Source: PLoS One. 2010 Oct 11;5(10):e13261. doi: 10.1371/journal.pone.0013261 (PMC2952585; doi:10.1371/journal.pone.0013261)
Supplement: Appendix S3 — Genotyping (0.04 MB DOC) [file pone.0013261.s003.doc]

**Supporting Information Appendix S3 Genotyping**

In KOALA, genomic DNA was extracted from buccal swabs using standard methods. DNA was amplified by using REPLI-g UltraFast technology (Qiagen™) as described previously.^S1^ In LISA, genomic DNA was extracted from EDTA blood. Five variants of the *FADS1 FADS2* gene cluster (rs174545, rs174546, rs174556, rs174561, rs3834458) were typed, which have been previously shown to be in strong linkage disequilibrium (LD) with each other.^S2,S3^ SNPs in both the KOALA and LISA study were selected based on previous publications in adult populations.^S2,S3,S4^

In addition, applying the tagger server program (<http://www.broadinstitute.org/mpg/tagger/>) in combination with HapMap we found that with the 3 SNPs rs174545, rs174546, rs174556 we could tag 27 SNPs between basepair positions 61234329 and 61372379 of *FADS1 FADS2*. The efficiency was 10.7 fold even though the two further SNPs rs174561 and rs3834458 could not be included as these are not included in the hapmap database. Genotyping of single nucleotide polymorphisms (SNPs) was realized with the iPLEX (Sequenom, San Diego, CA, USA) method by means of matrix assisted laser desorption ionization-time of flight mass spectrometry method (MALDI-TOF MS, Mass Array; Sequenom) in one laboratory according to the manufacturer’s instructions. Standard genotyping quality control included 10% duplicate and negative samples. Genotyping discordance rate was below 0.3%.

S1 Bottema RW, Reijmerink NE, Kerkhof M, Koppelman GH, Stelma FF, et al. ( 2008) Interleukin 13, CD14, pet and tobacco smoke influence atopy in three Dutch cohorts: the allergenic study. Eur Respir J; 32:593-602.

S2 Rzehak P, Heinrich J, Klopp N, Schaeffer L, Hoff S, et al. (2009) Evidence for an association between genetic variants of the fatty acid desaturase 1 fatty acid desaturase 2 ( FADS1 FADS2) gene cluster and the fatty acid composition of erythrocyte membranes. Br J Nutr; 101:20-6.

S3 Schaeffer L, Gohlke H, Muller M, Heid IM, Palmer LJ, et al. (2006) Common genetic variants of the FADS1 FADS2 gene cluster and their reconstructed haplotypes are associated with the fatty acid composition in phospholipids. Hum Mol Genet; 15:1745-56.

S4 Malerba G, Schaeffer L, Xumerle L, Klopp N, Trabetti E, et al. (2008) SNPs of the FADS gene cluster are associated with polyunsaturated fatty acids in a cohort of patients with cardiovascular disease. Lipids; 43:289-99.
