# Supplementary material for: Variants of the FADS1 FADS2 Gene Cluster, Blood Levels of Polyunsaturated Fatty Acids and Eczema in Children within the First 2 Years of Life
Source: PLoS One. 2010 Oct 11;5(10):e13261. doi: 10.1371/journal.pone.0013261 (PMC2952585; doi:10.1371/journal.pone.0013261)
Supplement: Appendix S4 — Determination of infants' IgE (0.04 MB DOC) [file pone.0013261.s004.doc]

**Supporting Information Appendix S4 Determination of infants’ IgE**

In the KOALA study total and specific IgE were determined at age 1 and 2 years.^S1^ Home visits were made at 1 year postpartum to collect capillary blood spots of the infants.. Total IgE and specific IgE against hen’s eggs, cow’s milk, and peanuts were measured as described earlier^S2,S3^ with modifications to accommodate the use of capillary blood samples.^S4^ The detection limit for total and specific IgE was <0.15 IU/ml and <0.36 IU/ml, respectively. Venous blood samples were collected by home visits 2 years post-partum and stored at -80^o^C until analysis. Samples were analysed for total IgE and specific IgE against hen’s eggs, cow’s milk, peanut, birch pollen, grass pollen, cat, dog and house dust mite using RAST as described earlier.^S5^ The detection limit for total and specific IgE was 0.50 and 0.10 IU/mL, respectively. All analyses were done by Sanquin Research (Amsterdam, NL).

In the LISA study blood samples were collected during physical examination of the infant at age 2 years and analysed for total and specific IgE using the RAST FEIA CAP system (Pharmacia, Freiburg, Germany) as previously described.^S6^

S1 Reimerink J, Stelma F, Rockx B, Brouwer D, Stobberingh E, et al. (2009) Early-life rotavirus and norovirus infections in relation to development of atopic manifestation in infants. Clin Exp Allergy; 39:254-60.

S2 Aalberse RC, Koshte V, Clemens JG (1981) Immunoglobulin E antibodies that crossreact with vegetable foods, pollen, and Hymenoptera venom. J Allergy Clin Immunol; 68:356-64.

S3 Stallman PJ, Aalberse RC (1977) Estimation of basophil-bound IgE by quantitative immunofluorescence microscopy. Int Arch Allergy Appl Immunol; 54:9-18.

S4 Stapel SO, Eysink PE, Vrieze J, Aalberse RC (2004) IgE testing in capillary blood. Pediatr Allergy Immunol; 15:230-3.

S5 Akkerdaas JH, Wensing M, Asero R, Fernandez RM, Knulst AC, et al. (2005) IgE binding to pepsin-digested food extracts. Int Arch Allergy Immunol; 138:203-8.

S6 Chen CM, Rzehak P, Zutavern A, Fahlbusch B, Bischof W, et al. (2007) Longitudinal study on cat allergen exposure and the development of allergy in young children. J Allergy Clin Immunol; 119:1148-55.
