# Supplementary material for: Variants of the FADS1 FADS2 Gene Cluster, Blood Levels of Polyunsaturated Fatty Acids and Eczema in Children within the First 2 Years of Life
Source: PLoS One. 2010 Oct 11;5(10):e13261. doi: 10.1371/journal.pone.0013261 (PMC2952585; doi:10.1371/journal.pone.0013261)
Supplement: Appendix S5 — Definition of outcome variable parental reported eczema (0.04 MB DOC) [file pone.0013261.s005.doc]

**Supporting Information Appendix S5 Definition of outcome variable parental reported eczema**

In both the KOALA and LISA study the definition of “parental reported eczema” is based on the questionnaire reported occurrence of “itchy rash that was coming and going” at any time within the first two years of life.

In the KOALA study the parents were asked at 7, 12, and 24 months’ postpartum “Has your child ever had an itchy rash that was variably present ..?” This was asked referring to the periods between questionnaires: “present ever?” (in the 7 months questionnaire)/ “present in the last 5 months” (in the 12 months questionnaire)/ “present in the second year of life?” (in the 24 months questionnaire). When this question was answered affirmatively in at least one of the questionnaires, the infant was defined as having eczema in the first 2 years of life. The infant was classified as having no eczema if the question was negative in all three questionnaires (or in two questionnaires when one questionnaire was missing), or when only diaper rash, rash around the eyes and/or scalp scaling was reported.^S1^

In the LISA study the definition is a combination of four related questions. Parents were asked at 6-, 12-, 18- and 24 months’ postpartum “1. Has your child had rash at some place other than the diaper area in the last 6 months?”, “2. How long did this rash last?”, “3. Did the rash occur repeatedly?”, and “4. Was this rash accompanied by itching”. Infants were defined as having developed eczema in the first 2 years of life, when questions 1 and 4 were answered affirmatively in any of the questionnaires and either it was approved that this itchy rash occurred repeatedly or lasted more than 14 days.^S2^

S1 Snijders BE, Thijs C, van Ree R, van den Brandt PA (2008) Age at first introduction of cow milk products and other food products in relation to infant atopic manifestations in the first 2 years of life: the KOALA Birth Cohort Study. Pediatrics; 122:e115-e122.

S2 Chen CM, Sausenthaler S, Bischof W, Herbarth O, Borte M, et al. (2009) Perinatal exposure to endotoxin and the development of eczema during the first 6 years of life. Clin Exp Dermatol; Epub ahead of print doi:10.1111/j.1365-2230.2009.03460.x.
