# Supplementary material for: Variants of the FADS1 FADS2 Gene Cluster, Blood Levels of Polyunsaturated Fatty Acids and Eczema in Children within the First 2 Years of Life
Source: PLoS One. 2010 Oct 11;5(10):e13261. doi: 10.1371/journal.pone.0013261 (PMC2952585; doi:10.1371/journal.pone.0013261)
Supplement: Appendix S6 — Statistical analysis (0.04 MB DOC) [file pone.0013261.s006.doc]

**Supporting Information Appendix S6 Statistical analysis**

Allele frequencies and Hardy-Weinberg Equilibrium (HWE) for the study population and for both studies separately were conducted with procedure "proc allele" of the statistical software module SAS/GENETICS of SAS version 9.1.3. HWE was tested by Fisher’s exact test. To examine linkage disequilibrium (LD), Lewontin’s D’ and pairwise squared correlations r^2^ were calculated by the software JLIN.^S1^

Normal distribution of fatty acids was tested using Kolmogorov-Smirnov-tests and evaluated by Q-Q-plots and stem-and-leaf-plots (procedure "proc univariate" of SAS/STAT 9.1.3). Severely right-skewed outcomes like gamma-linolenic acid (C18:3n-6), alpha-linolenic acid (C18:3n-3), eicosapentaenoic acid (C20:5n-3) and docosahexaenoic acid (C22:6n-3), and total and specific IgE were natural log-transformed.

Single SNP linear regression analyses for the relation between *FADS* variants and the nine continuous outcome variables (fatty acids) were conducted applying an additive model where homozygous minor allele carriers were coded as 2, heterozygous coded as 1, and homozygous major allele carriers coded as 0 (= reference category).

To keep the nominal level of type 1 error (false positive results) to 5% despite numerous tests of associations, *P*-values of the primary analyses were conservatively corrected by Bonferroni correction; i.e. multiplying the uncorrected *P*-values by the number of analyzed outcomes times the number of analyzed SNPs (i.e. 45).

Logistic regression was applied to evaluate the effect of each single SNP and of each fatty acid separately on the dichotomous coded outcome “parental reported eczema during the first two years of life”. The potential associations of the SNPs were modelled by including two indicator coded dummies for the homozygous and heterozygous minor allele carriers (reference is homozygous major allele carriers). Confidence intervals (95%-CI) of the respective odds ratios (OR) were used to assess associations between genotypes and child’s eczema. In addition, *P*-values for test of multiplicative allelic trend were calculated by applying logistic regression modelling coding the SNPs as 0, 1, 2 (1 degree of freedom (df)).

Analyses were performed without and with adjustment for study cohort (indicator coded dummy variables: KOALA study conventional, KOALA study alternative recruitment group vs. LISA study as reference), sex, maternal education, maternal smoking during pregnancy, exclusive breastfeeding for at least 3 months, but not for family history of atopy (asthma or allergy) to avoid overcorrection as the *FADS* gene cluster may be related to atopy in parents. No corrections for multiple testing were applied in logistic regression.

In addition, we investigated potential interactions between studies and SNPs and studies and PUFAs for the outcome eczema by performing separate analyses for the KOALA und the LISA studies to evaluate potential influences of different populations and study designs on results.

All analyses were performed using the statistical software SAS, version 9.1.3 (SAS/STAT, SAS/GENETICS) ^S2,S3^, except calculation of linkage disequilibrium measures, which were computed by the software JLIN.^S1^

S1 Carter KW, McCaskie PA, Palmer LJ (2006) JLIN: a java based linkage disequilibrium plotter. BMC Bioinformatics; 7:60.

S2 SAS Institute Inc. 2004. SAS/STAT 9.1 User's Guide. SAS Institute Inc., Cary, NC, USA, 2004.

S3 SAS Institute Inc. 2005. SAS/Genetics 9.1.3 Users's Guide. Cary, NC: SAS Insdtitute Inc., 2005.
