# Supplementary material for: Variants of the FADS1 FADS2 Gene Cluster, Blood Levels of Polyunsaturated Fatty Acids and Eczema in Children within the First 2 Years of Life
Source: PLoS One. 2010 Oct 11;5(10):e13261. doi: 10.1371/journal.pone.0013261 (PMC2952585; doi:10.1371/journal.pone.0013261)
Supplement: Table S1 — Characteristics of the five analyzed variants in the FADS1 FADS 2 gene region (0.37 MB DOC) [file pone.0013261.s007.doc]

**Supporting Information Table S1.** Characteristics of the five analyzed variants in the *FADS1 FADS 2* gene region

Note: Z indicates deletion; SNP build 130 accessed 23 Nov 2009, Map to Genom Build 36.3
