# Supplementary material for: Variants of the FADS1 FADS2 Gene Cluster, Blood Levels of Polyunsaturated Fatty Acids and Eczema in Children within the First 2 Years of Life
Source: PLoS One. 2010 Oct 11;5(10):e13261. doi: 10.1371/journal.pone.0013261 (PMC2952585; doi:10.1371/journal.pone.0013261)
Supplement: Table S4 — Mean levels of fatty acids (PUFA) by genotype of five variants in the FADS1 FADS 2 gene region in the LISA study (0.39 MB DOC) [file pone.0013261.s010.doc]

**Supporting Information Table S4.** Mean levels of fatty acids (PUFA) by genotype of five variants in the *FADS1 FADS 2* gene region in the LISA study

Note: Mean levels of GLA, ALA, EPA and DHA are naturally logged means to account for the severely skewed distributions of these fatty acids.
