# Supplementary material for: Variants of the FADS1 FADS2 Gene Cluster, Blood Levels of Polyunsaturated Fatty Acids and Eczema in Children within the First 2 Years of Life
Source: PLoS One. 2010 Oct 11;5(10):e13261. doi: 10.1371/journal.pone.0013261 (PMC2952585; doi:10.1371/journal.pone.0013261)
Supplement: Table S5 — Associations of the five analyzed variants in the FADS1 FADS 2 gene region with fatty acids in the KOALA study (0.39 MB DOC) [file pone.0013261.s011.doc]

**Supporting Information Table S5.** Associations of the five analyzed variants in the *FADS1 FADS 2* gene region with fatty acids in the KOALA study

Note: Corrected *P*-values to account for multiple testing are obtained by dividing not corrected *P*-values by 45 (5 single SNP-analyses of 9 outcomes);

ß‑coeffcient is the respective regression estimate of the additive coded SNP (0/1/2) for the respective outcome (PUFA) in a simple regression model.
