# Supplementary material for: Variants of the FADS1 FADS2 Gene Cluster, Blood Levels of Polyunsaturated Fatty Acids and Eczema in Children within the First 2 Years of Life
Source: PLoS One. 2010 Oct 11;5(10):e13261. doi: 10.1371/journal.pone.0013261 (PMC2952585; doi:10.1371/journal.pone.0013261)
Supplement: Table S7 — Association of FADS1 FADS 2 variants with parental reported eczema (0.38 MB DOC) [file pone.0013261.s013.doc]

**Supporting Information Table S7.** Association of *FADS1 FADS 2* variants with parental reported eczema

Note: Odds ratios (OR) of indicator coded SNPs (reference is homozygous major alleles genotype) on eczema are estimated by logistic regression. Adjustment comprises sex, maternal education, maternal smoking during pregnancy and exclusive breastfeeding for at least 3 months in both cohort stratified analyses. The KOALA-study was in addition adjusted for recruitment group (conventional vs. alternative recruitment group).
