# Supplementary material for: Variants of the FADS1 FADS2 Gene Cluster, Blood Levels of Polyunsaturated Fatty Acids and Eczema in Children within the First 2 Years of Life
Source: PLoS One. 2010 Oct 11;5(10):e13261. doi: 10.1371/journal.pone.0013261 (PMC2952585; doi:10.1371/journal.pone.0013261)
Supplement: Table S8 — Association of fatty acids with parental reported eczema (0.38 MB DOC) [file pone.0013261.s014.doc]

**Supporting Information Table S8.** Association of fatty acids with parental reported eczema

Note: Odds ratios (OR) of measured PUFA on eczema are estimated by logistic regression. Adjustment comprises sex, maternal education, maternal smoking during pregnancy and exclusive breastfeeding for at least 3 months in both cohort stratified analyses. The KOALA-study was in addition adjusted for recruitment group (conventional vs. alternative recruitment group).
